# Inner pace: A dynamic exploration and analysis of basketball game pace

**DOI:** 10.1371/journal.pone.0320284

**Published:** 2025-05-12

**Authors:** Fei Zhang, Qing Yi, Rui Dong, Jin Yan, Xiao Xu

**Affiliations:** 1 Hangzhou Normal University, Hangzhou, Zhejiang, China; 2 College of Physical Education, Dalian University, Dalian, Liaoning, China; 3 College of Physical Education, South China Normal University, Guangzhou, China; 4 School of Physical Education and Sports Science, Soochow University, Suzhou, China; Universidade Federal de Goias, BRAZIL

## Abstract

This study aims to investigate the dynamics of basketball game pace and its influence on game outcomes through a novel intra-game segmentation approach. By employing K-means clustering on possession duration, we categorized possessions from 1,141 NBA games in the 2019–2020 season into high-frequency (HFS), low-frequency (LFS), and normal-frequency segments (NFS). A sliding window method was utilized to identify these segments, revealing distinct temporal patterns within games. To analyze the predictive value of these segments, we applied machine learning models, including Random Forest and Light Gradient Boosting Machine (LightGBM), complemented by SHapley Additive exPlanations (SHAP) for interpretability. Our findings demonstrate that HFS segments increase toward the end of each quarter, driven by rapid transitions and tactical urgency, whereas LFS segments dominate the middle phases, reflecting strategic tempo control. NFS accounts for the majority of game time but decreases as the game progresses. The LightGBM analysis highlighted the importance ranking of key performance indicators (KPIs) across different segments and revealed differences in the importance of these indicators within each segment. Compared to traditional methods, our approach provides a finer-grained analysis of game pace dynamics and offers actionable insights for optimizing coaching strategies. This study not only advances the understanding of basketball game rhythm but also establishes a robust framework for integrating machine learning and statistical models in sports analysis.

## Introduction

Basketball is a sport characterized by frequent transitions between offense and defense and intense physical confrontations. Over the course of 40 or 48 minutes, both teams engage in a comprehensive competition involving physical endurance, skills, tactics, and psychological resilience, all aimed at gaining a competitive edge and ultimately securing victory [1]. Performance analysis in sports systematically collects and analyzes athletic data to assess and improve the performance of athletes or teams [2]. Given basketball’s complexity and competitive nature, extracting valuable insights from competition data poses significant challenges [3,4].

Previous studies have utilized key performance indicators (KPIs) to distinguish between winning and losing teams, aiming to evaluate team performance, optimize competition strategies, and predict match outcomes [5]. Scholars have noted that the endogeneity of the sport complicates quantitative research, as the system constantly evolves, influenced by both internal and external environments, which causes KPIs to change continuously [3]. Consequently, research on KPIs in basketball has introduced contextual factors to balance the impact of this endogeneity.

Contextual factors related to basketball performance analysis include game phase (e.g., crucial moments and quarters), opponent quality, home and away status, game pace, external disturbances (e.g., referee decisions, injuries, crowd interference), and more [6–10]. Among these, game pace is an important contextual factor indicator. Introduced by Oliver Deen, it is based on the concept of possessions and emphasizes measuring the speed of the game through the number of offensive and defensive transitions in a match [1].

Earlier research classified games or quarters into fast-paced, normal-paced, and slow-paced categories based on the number of possessions for the entire game or quarter, subsequently identifying KPIs [9,11]. Later studies analyzed game pace from various perspectives rather than merely categorizing games by pace. For instance, Scanlan et al. [12] compared league paces by game duration, suggesting that 40-minute games tend to have a slower pace compared to 48-minute games, and that the ability of KPIs to distinguish winning teams and predict outcomes diminishes with shorter game durations. Courel-Ibáñez and McRobert [13] proposed that actively manipulating game pace can serve as a viable strategy for coaches and teams seeking a competitive advantage. Ibañez et al. [14] argued that a faster game pace, driven by changes in basketball rules, enhances the game’s entertainment value, thereby attracting more fans. Russell et al. [15] examined dynamic changes in players’ external demands based on game pace.

Given the dynamic complexity of basketball, this study aims to refine the granularity of game pace research to the level of intra-game segments, addressing the following three questions:

(1) Are there high-frequency, low-frequency, and normal-frequency possession-transition segments during a game when sliced a game according to possession duration?
(2) Do these high-frequency and low-frequency possession segments exhibit specific temporal characteristics within a game?
(3) Which statistics in these three different segments can better predict game outcomes, providing training and strategic guidance for coaches and players?

## Materials and methods

### Samples

The data for this study were obtained from publicly available resources on the professional sports analysis website ESPN. Through data scraping, play-by-play data for a total of 1,143 NBA games from the 2019–2020 season were collected. Two games were excluded due to the data completeness, resulting in a total of 1,141 games included in the analysis. To ensure consistency, play-by-play data from overtime periods were not considered. Play-by-play data refer to detailed records of every action and event that occurs during a game [16]. This data typically includes timestamps and event descriptions to accurately track the game’s progress. In this study, the play-by-play data primarily includes the following components:

- GameID: Contains the game type and a unique identifier for each game.
- Home/Away Teams: Identifies the teams participating in the game.
- Home Team Events: Describes specific events that occurred for the home team, such as shots, free throws, turnovers, fouls, assists, and rebounds.
- Away Team Events: Describes specific events that occurred for the away team.
- Player: Identifies the players involved in the events.
- Timestamp: Records the specific time at which events occurred, measured in seconds.
• Game Status: Captures the score, game period (e.g., first quarter, second quarter), and remaining time at the time of each event.

#### Data reliability.

In this study, the intra-class correlation coefficient (ICC) was used to reflect the reliability of the data [17]. Sample of 20 games was selected from the 1,141 games by random sampling. Four analysts with professional basketball analysis experience conducted a detailed consistency check of the variables by combining basketball game videos with the play-by-play data. The final results showed that the intra-class correlation coefficient (ICC) was 1.00, indicating the validity of the consistency check and the reliability of the data source.

### Data processing

#### Identifying game possession segments.

This study first identifies the start and end times of each possession, calculates the duration of each possession, and then clusters the possession duration into fast, slow, and normal categories. Using the thresholds defined by these three categories, a sliding window analysis is applied to continuous possession events to delineate specific game frequency segments. To ensure that each segment unit provides sufficient information for subsequent analysis, the minimum threshold for continuous possession events is set to 5.

(1) Clustering Fast, Slow, and Average Possessions

**Fig 1:**
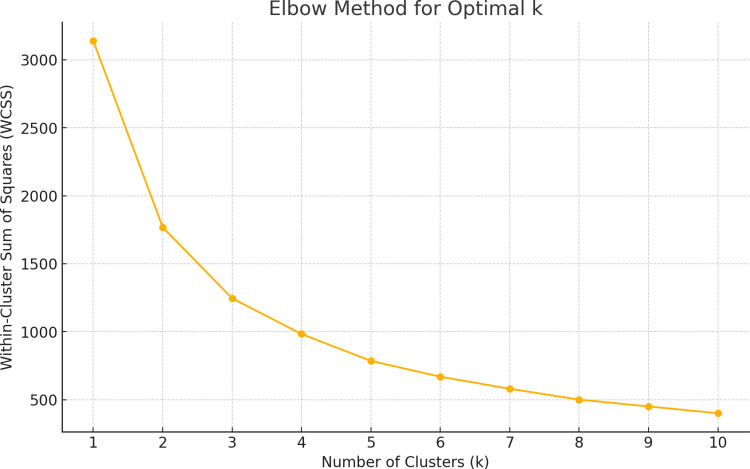
Optimal number of clusters determined by the elbow method.

In basketball, the term “possession” refers to the period during which a team controls the ball until the opposing team regains it [1]. In the play-by-play data, the number and duration of possessions are determined by counting possession-ending events for both the home and away teams and identifying the corresponding start and end events. Subsequently, the K-means method was applied to cluster possession durations, ensuring that different possessions were categorized based on their duration. K-means clustering is a classic and efficient clustering algorithm, particularly excelling in handling large-scale datasets. Before applying the clustering, this study employed the Elbow Method to determine the optimal number of clusters (k). As shown in Fig 1, the Within-Cluster Sum of Squares (WCSS) curve begins to level off at k = 3, indicating that the marginal benefit of increasing the number of clusters significantly diminishes beyond this point. Therefore, k = 3 was selected as the optimal number of clusters for our analysis. This choice ensures both the validity of the model and the interpretability of the results. After these calculations, all possession durations are clustered using the K-means method to classify them into possession types based on distinct centroids [18]. The statistical data and K-means results are presented in Table 1.

**Table 1:** K-means results.

Statistics		K-means Results			
Average Possessions per Game	205.19 ± 10.86		Sample Size (Average)	Possession Duration Range	Average Possession Duration
Possession Range	174-249	Cluster1 (fast)	43.83	0.00-8.00	4.99 ± 2.65
Possession Duration	0-63	Cluster2 (normal)	111.76	9.00-18.00	13.43 ± 2.75
Possession Time Range	13.92 ± 6.77	Cluter3 (slow)	49.6	＞18.00	21.88 ± 3.90

(2) Setting Thresholds and Dividing the Game into Different Segments

Based on the research objective, we attempted to use fixed time thresholds (derived from clustering results) to distinguish different game segments. We acknowledge that event sequences (e.g., turnovers, missed shots) also play a role in shaping game pace. However, we chose possession duration over event sequences as the threshold because it serves as a higher-level aggregate indicator that inherently reflects the combined effects of these micro-level events. For example, shorter possession durations are often associated with turnovers or fast breaks, while longer possession durations may indicate more organized offensive plays.

At the same time, the use of fixed thresholds is based on data-driven, objective results, ensuring generalizability, interpretability, and practical applicability. Although different teams may exhibit variations in playstyle, our research framework aims to uncover the general patterns of game pace rather than the specific characteristics of individual teams.

Based on the K-means results, the range of normal possession durations is 9–18 seconds. Therefore, this study uses this result as a threshold and applies the sliding window technique to classify continuous possession events of more than 5 into high-frequency possession transition segments (HFS), low-frequency possession transition segments (LFS), and normal-frequency possession transition segments (NFS). The concepts are as follows:

- HFS Threshold: Average possession duration < 9 seconds over a sequence of 5 or more possessions.
- LFS Threshold: Average possession duration > 18 seconds over a sequence of 5 or more possessions.

(3) Using Sliding Window Analysis to Determine the Segment Classification of Different Possessions During the Game

Process the data for each game individually to ensure that calculations do not overlap between different games.

- Initial Settings Define the high-frequency threshold as $T_{high}$ = 9 seconds

Define the low-frequency threshold as $T_{low\;}$= 18 seconds

Define the minimum sequence length as N= 5

- Sliding Window Calculation Using the sliding window method [19,20], calculate the average possession time for N consecutive possession events.

For the starting position $\begin{array}{c} i \end{array}$:


$$Average\;possession\;time=\frac{1}{N}\sum_{j=i}^{i+N-1}P_{j}$$

where $P_{j}$ represents the possession time for the j-th event.

- HFS Determination Starting from event  i, if the condition is met:

$$\frac{1}{N}\sum_{j=i}^{i+N-1}P_{j}\leq T_{high}$$

mark the possessions within that window as HFS.

If the condition is met, extend the window until the condition is no longer met:


$$\frac{1}{N+k}\sum_{j=i}^{i+N+k-1}P_{j}>T_{high}$$

where k is the number of additional possessions.

- LFS Determination Starting from event  i, if the condition is met:

$$\frac{1}{N}\sum_{j=i}^{i+N-1}P_{j}\geq T_{low}$$

mark the events within that window as LFS.

If the condition is met, extend the window until the condition is no longer met:


$$\frac{1}{N+k}\sum_{j=i}^{i+N+k-1}P_{j}{<T}_{low}$$

where k is the number of additional possessions.

Additionally, other possessions are classified as NFSs.

Finally, all game possessions are classified into segments, and the information from the three types of segments is integrated and presented in charts and visualizations.

#### Predicting temporal features of each frequency segment based on random forest.

To further study the temporal features of the three types of game frequency segments, we divided the 48-minute, 4-quarter basketball game into 16 periods, each 3 minutes long. Subsequently, the distribution of the three types of game frequency segments within the 16 periods of each game was set as feature variables, and machine learning was employed for prediction. Due to its robustness and ability to handle nonlinear relationships, we chose the random forest regression model [21,22].

The random forest regression model consists of multiple decision trees, each trained by minimizing the mean squared error (MSE). The model uses 100 estimators, with each tree’s prediction value being ${\hat{y}}_{i}^{\left(t\right)}$, where t represents the prediction of the t-th tree. The final prediction model of the random forest is the average of all tree predictions:


$${\hat{y}}_{i}=\frac{1}{T}\sum_{t=i}^{T}{\hat{y}}_{i}^{\left(t\right)}$$

Finally, a new test set was created, containing the 1–16 segments of a 48-minute game. The established random forest model was used to make predictions on this test set, and the prediction results were visualized.

#### Determine the importance of different segments indicators.

This section attempts to determine the specific indicator values that influence the results of score within different frequency segments and their impact. Based on previous studies, team performance metrics and some coaching intervention metrics were extracted from the play-by-play data. Additionally, the partial scores of both team in the segments, and the number of possessions were extracted. Table 2 presents the definitions and information of these variables. All variables were normalized by the number of possessions and incorporated into the prediction model in the form of 100 possessions [3].

**Table 2:** Definition of variables.

Variable	Abbreviation	Definition
Two-point Made	2-ptM	Successfully made a two-point field goal from inside the three-point line.
Two-point Miss	2-ptm	Missed a two-point field goal from inside the three-point line.
Three-point Made	3-ptM	Successfully made a three-point field goal from outside the three-point line.
Three-point Miss	3-ptm	Missed a three-point field goal from outside the three-point line.
Free Throw Made	FTM	Successfully made a free throw from the free-throw line.
Free Throw Miss	FTm	Missed a free throw from the free-throw line.
Assist	AST	A pass to a teammate that directly leads to a score.
Defensive Rebound	DREB	A rebound by the defensive team after an opponent’s missed shot.
Offensive Rebound	OREB	A rebound by the offensive team after their own missed shot.
Turnover	TOV	Losing possession of the ball to the opposing team.
Steal	STL	Successfully taking the ball away from the opponent.
Block	BLK	Deflecting an opponent’s field goal attempt, preventing a score.
Personal Foul	PF	A breach of the rules that concerns illegal personal contact with an opponent.
Technical Foul	TF	A foul for unsportsmanlike behavior or other infractions not involving physical contact.
Unsportmanlike Foul	UF	A severe foul involving excessive physical contact.
Violation	V	A breach of the basic rules of basketball (e.g., traveling, double dribble).
Timeout	TOT	A stoppage in play requested by the coach to discuss strategy or rest.
Substitution	SUB	Replacing one player with another during the game.
Team Status	HA	Team status indicates whether the target team is the home team or the away team.

LightGBM (Light Gradient Boosting Machine) is a decision tree-based gradient boosting framework designed to minimize a specific loss function L. The objective function consists of a loss term measuring the model’ s prediction error and a regularization term controlling the complexity of the model:


$$L=\sum_{i=1}^{n}l(y_{i},{\hat{y}}_{i})+\Omega (f)$$

Where $l(y_{i},{\hat{y}}_{i})$ represents the loss function, which measures the difference between the predicted value ${\hat{y}}_{i}$ and the actual value $y_{i}.\;\Omega (f)$ is the regularization term to prevent overfitting by limiting the complexity of the decision tree f.

During each boosting iteration t, LightGBM updates the prediction model as follows:


$${\hat{y}}_{i}^{\left(t\right)}={\hat{y}}_{i}^{\left(t-1\right)}+f_{t}(x_{i})$$

Where $f_{t}(x_{i})$ is the new weak learner (decision tree) added at iteration t. To optimize the model, the new decision tree $f_{t}$ minimizes the following second-order approximation of the loss:


$$L^{\left(t\right)}=\sum_{i=1}^{n}g_{i}f_{t}(x_{i})+\frac{1}{2}h_{i}f_{t}^{2}(x_{i})+\Omega (f_{t})$$

Where:

$g_{i}=\frac{\partial l({\hat{y}}_{i},{{\hat{y}}_{i}}^{\left(t-1\right)})}{\partial {{\hat{y}}_{i}}^{\left(t-1\right)}}$ is the first-order gradient of the loss function.

$h_{i}=\frac{\partial^{2}l({\hat{y}}_{i},{{\hat{y}}_{i}}^{\left(t-1\right)})}{\partial {{\hat{y}}_{i}}^{{\left(t-1\right)}^{2}}}$ is the secvond-order gradient (Hessian) of the loss function.

We use LightGBM as the core machine learning model for prediction, establishing three models for HFS, NFS, and LFS. The independent variables are the standardized data metrics from Table 2 for different segments of each team, and the dependent variable is the net point difference in each segment. The support for categorical features and the ability to control complexity in LightGBM enable high prediction accuracy and effectively prevent overfitting [23]. As a result, it is a powerful algorithm widely used in the field of machine learning.

However, LightGBM has is a black-box model, which brings certain difficulties to model interpretation [23]. Therefore, SHapley Additive exPlanations (SHAP), based on cooperative game theory, is chosen to interpret the LightGBM model [24]. The SHAP-based LightGBM model ensures intuitive interpretability at both global and local levels, guaranteeing the interpretation and determination of feature importance in the model results [25].

The Shapley value for a feature j is computed as:


$$\phi_{j}=\;\sum_{S\subseteq N/\{j\}}\frac{\left|S|!\cdot (|N|-|S|!\right)}{|N|!}\cdot [f(S\cup \{j\})-f(S)]$$

Where:

$\phi_{j}$ is the Shapley value for feature j, indicating its contribution to the prediction.

N is the set of all features.

S is a subset of features excluding j.

f(S) is the model’s prediction using only the features in subset S.

By combining LightGBM and SHAP, this study achieved both high prediction accuracy and intuitive model interpretation. LightGBM provided robust predictions, while SHAP made the model’s decision-making process transparent. SHAP identified key performance indicators (KPIs) that were most influential across different frequency segments, offering insights into their varying impacts.

## Result

### Descriptive analysis of different frequency segments

Using sliding window analysis, this study classified all possessions occurring in each game into three different frequency segments: high-frequency possession transition segments, low-frequency possession transition segments, and normal-frequency possession transition segment. The HFS includes more than five consecutive possession events with an average possession duration of less than 9 seconds, indicating a faster pace during this segment. The LFS includes more than five consecutive possession events with an average possession duration of more than 18 seconds, indicating a slower pace during this segment. Possessions not fitting into these categories were classified as the NFS, which did not exhibit continuous fast or slow pace characteristics. Table 3 provides a descriptive analysis of the high- and low-frequency segments presented on a per-game basis.

**Table 3:** Descriptive analysis result of different frequency segments.

	Game Phase	Number of Occurrences	Total Duration	Duration per Occurrence	Number of Possessions	Possessions per Occurrence
HFS	Entire Game	4.32 ± 2.04	242.63 ± 130.18	56.21 ± 24.36	29.03 ± 15.25	6.73 ± 2.81
	First Quarter	1.49 ± 0.68	81.38 ± 44.09	54.78 ± 19.00	9.52 ± 5.09	6.41 ± 2.14
	Second Quarter	1.61 ± 0.81	91.94 ± 55.85	56.97 ± 25.99	10.78 ± 6.45	6.68 ± 2.91
	Third Quarter	1.58 ± 0.81	91.89 ± 57.68	58.03 ± 27.34	10.76 ± 6.64	6.79 ± 3.07
	Fourth Quarter	1.62 ± 0.81	88.82 ± 52.81	54.95 ± 23.48	11.22 ± 6.38	6.94 ± 2.89
LFS	Entire Game	6.56 ± 2.27	876.82 ± 364.65	133.63 ± 72.86	47.15 ± 19.61	7.19 ± 3.97
	First Quarter	1.73 ± 0.80	212.64 ± 113.05	122.97 ± 53.67	11.48 ± 6.12	6.64 ± 2.93
	Second Quarter	1.87 ± 0.84	240.43 ± 129.74	128.66 ± 64.69	12.92 ± 6.99	6.91 ± 3.53
	Third Quarter	1.90 ± 0.87	254.97 ± 142.54	134.19 ± 73.70	13.68 ± 7.67	7.20 ± 4.01
	Fourth Quarter	2.01 ± 0.87	292.77 ± 150.74	145.56 ± 88.21	15.74 ± 8.13	7.82 ± 4.81
NFS	Entire Game	10.13 ± 2.12	1757.28 ± 334.22	173.51 ± 178.94	129.65 ± 24.41	12.80 ± 13.04
	First Quarter	2.55 ± 0.97	501.82 ± 194.43	196.55 ± 193.48	37.24 ± 14.19	14.58 ± 14.17
	Second Quarter	2.40 ± 0.96	454.00 ± 226.66	189.28 ± 194.11	33.48 ± 16.40	13.96 ± 14.17
	Third Quarter	2.48 ± 0.97	437.65 ± 215.92	176.55 ± 179.59	32.09 ± 15.48	12.95 ± 13.01
	Fourth Quarter	2.75 ± 1.00	373.45 ± 180.74	135.70 ± 140.60	27.56 ± 13.05	10.01 ± 10.21

**Fig 2:**
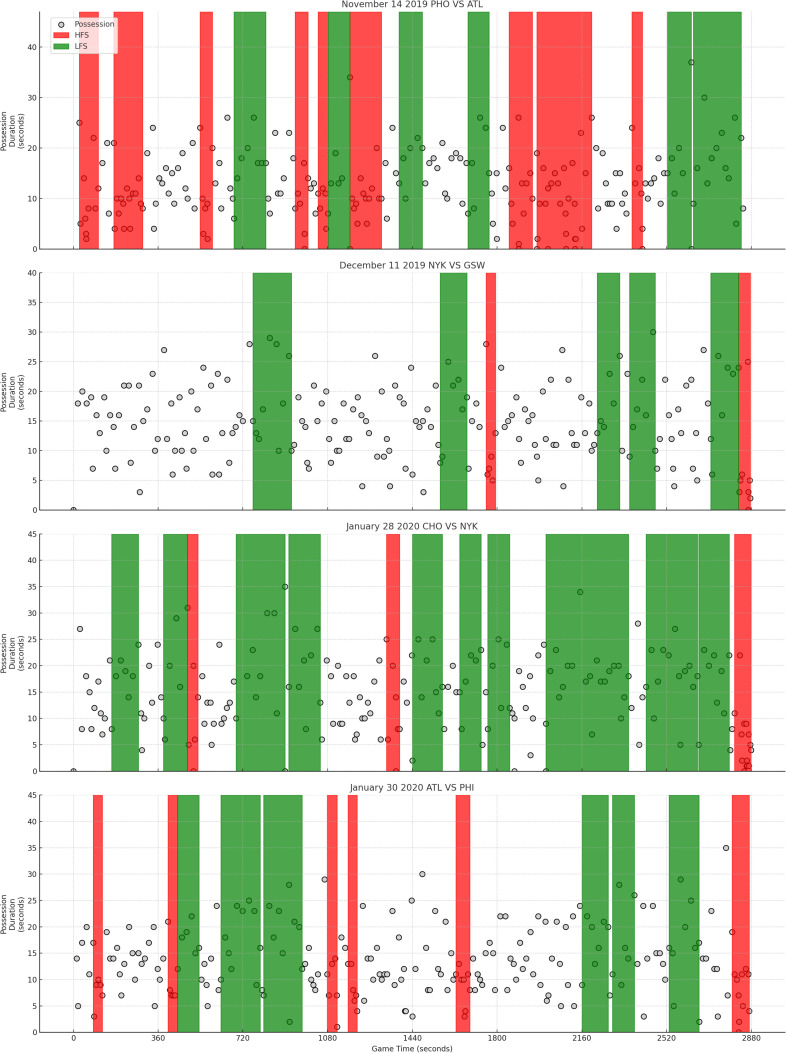
Examples of different frequency segments in games. (X-axis: Game time, in seconds. Y-axis: Possession duration, in seconds. Dots represent consecutive possession events of games. Red and green backgrounds represent the time periods belonging to HFS and LFS, respectively, while the white background represents NFS.).

For the high-frequency possession transition segment, it appears an average of 4.32 times each game, with over 29 possessions included in this segment on average. Due to its overall faster pace, the duration per occurrence are shorter compared to the other two segments. For the low-frequency possession transition segment, it appears an average of 6.56 times each game, with over 47 possessions included in this segment. The average duration of each occurrence is more than two minutes, with a total duration of over 14 minutes in a entire game. The normal possession transition segment is the main type in a game, with more than 129 possessions included in this segment within a game, indicating that most of the game time does not exhibit continuous fast or slow possession transitions. Fig 2 contains possession transition dynamic graphs for four example games with different segments distinguished by background colors.

### Random forest prediction results

Table 4 provides the prediction results of the random forest for the duration of the three types of frequency segments in each 3-minute game segment. To evaluate the generalization ability of the Random Forest regression model, 10-fold cross-validation was employed in this study. The resulting performance metrics were: Root Mean Squared Error (RMSE) = 0.484, Mean Absolute Error (MAE) = 0.408 ± 0.132, and R-squared (R²) = 0.44 ± 0.117. The R² value of 0.44 indicates that the model can explain 44% of the variance in the target variable. Although this value does not represent a high level of fit, it is considered a reasonable result for the research problem at hand. The overall durations are close to the descriptive analysis in Table 3. The duration range for the high-frequency possession transition segment is 9.45s-26.20s, for the low-frequency possession transition segment is 43.69s-77.11s, and for the regular possession transition segment is 89.16s-126.86s.

**Table 4:** Result of random forest.

Game Second	HFS	LFS	NFS
0-180	9.45	43.69	126.86
180-360	11.13	44.79	124.08
360-540	12.44	46.90	120.66
540-720	15.31	43.90	120.79
720-900	12.04	49.05	118.91
900-1080	13.59	53.03	113.39
1080-1260	15.85	53.34	110.80
1260-1440	20.00	46.25	113.75
1440-1620	13.47	57.34	109.19
1620-1800	14.78	56.97	108.25
1800-1980	15.48	59.01	105.51
1980-2160	19.01	53.58	107.41
2160-2340	13.41	62.95	103.64
2340-2520	13.31	72.58	94.12
2520-2700	12.43	77.11	90.46
2700-2880	26.20	53.70	89.16
overall	237.90	874.18	1756.99

**Fig 3:**
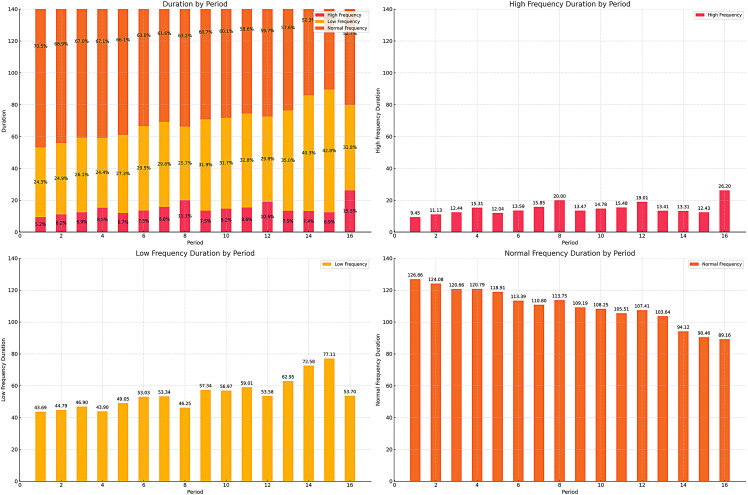
Random forest prediction results. (The X-axis represents 1-16 periods in the game, totaling 48 minutes of a regular match. The Y-axis represents the duration of the segments. The top-left bar chart shows the time proportion changes of the three types of segments across the 16 periods. The top-right, bottom-left, and bottom-right bar charts respectively represent the predicted time changes for HFS, LFS, and NFS.).

To better present the changes of the three frequency segments over the entire game, Fig 3 visualizes these results.

### SHAP results used to explain the LightGBM model

Models for three types of segment was built using the LightGBM method. The target value is the point difference between one team and the opponent in the given segment. The feature variables are the 19 indicators in Table 2, including regular team statistics, coach intervention indicators, and context factors. To comprehensively evaluate the predictive performance and generalization ability of the LightGBM model, we employed 10-fold cross-validation as the primary evaluation method. We assessed the model on three different datasets and obtained the following results. For Model 1 (HFS), the RMSE was 11.6393 ± 1.7986, the MAE was 8.3416 ± 1.1103, and the R² was 0.7790 ± 0.0419. Model 2 (NFS) showed an RMSE of 4.3767 ± 0.4427, an MAE of 3.3296 ± 0.3429, and an R² of 0.7723 ± 0.0230. Finally, Model 3 (LFS) produced an RMSE of 10.3692 ± 1.3494, an MAE of 7.6462 ± 0.8564, and an R² of 0.7289 ± 0.0385. Among the three models, NFS had the longest duration and the largest sample size, resulting in the smallest RMSE and MAE prediction errors. While the HFS and LFS models showed relatively larger errors, these were still within an acceptable range. All three models achieved R² values greater than 0.7, indicating strong explanatory power and suggesting that the models were able to accurately predict the target variable in most cases.

**Fig 4:**
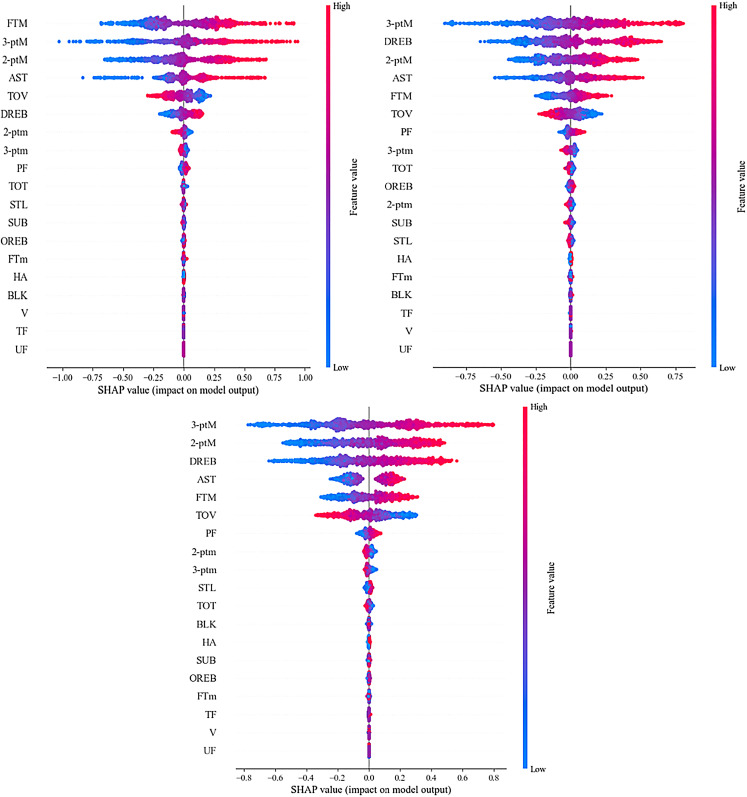
Feature importance of HFS, LFS and NFS. (Fig 4-1, Fig 4-2, and Fig 4-3 represent HFS, NFS, and LFS, respectively. From left to right are HFS, NFS, and LFS. The X-axis shows the magnitude of each feature’s impact on the prediction results. The farther a point is from the centerline (zero), the greater the feature’s impact on the model output. Positive SHAP values indicate a positive impact, while negative SHAP values indicate a negative impact. The Y-axis ranks the features by their influence from top to bottom, with the top features having a greater overall impact on the model output, and the bottom features having a lesser impact. Pink dots represent that the feature value has a positive impact on the model prediction in this observation. Blue dots indicate that the feature value has a negative impact on the model prediction in this observation.).

**Fig 5:**
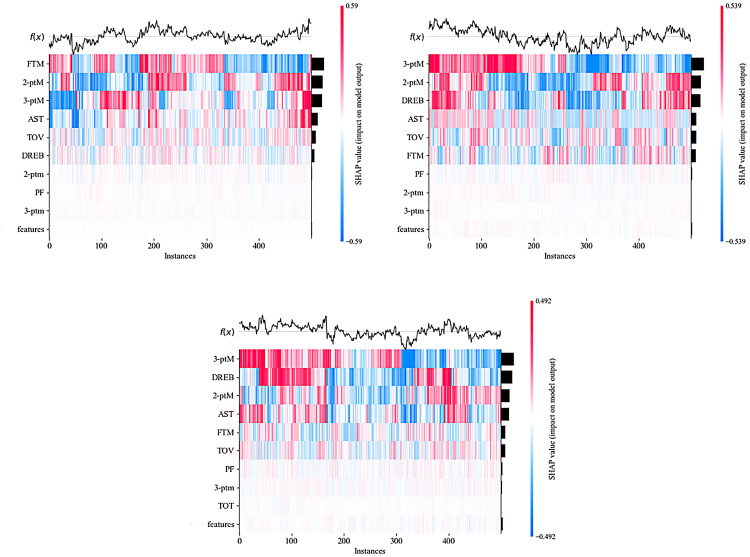
SHAP heatmap. (Fig 5-1, Fig 5-2, and Fig 5-3 represent HFS, NFS, and LFS, respectively. The left Y-axis shows the ranking of important features, and the right Y-axis visualizes these features. The color intensity in the image represents the magnitude of the SHAP values: the darker the color, the larger the absolute value of the SHAP, and the greater its impact on the model. The top part visualizes the model’s prediction results under these values. The chart visualizes data from 500 test set samples, as too much data would affect the clarity of the visualization.).

The results of quantifying the variable importance in the model using the SHAP method are shown in Table 5. The mean absolute value of the SHAP values is obtained by averaging the absolute importance values of a specific feature across all samples, representing the overall ranking of the feature’s importance. Fig 4 visualizes the ranking of feature variable importance in the three types of games. Fig 5 shows the importance of feature variables refined to individual games within the three types of matches (for visualization purposes, only 500 samples were used).

**Table 5:** Feature importance result of SHAP.

	HFS	LFS	NFS
DREB	0.0668	0.2339	0.2274
SUB	0.0033	0.0078	0.0023
FTM	0.2755	0.0883	0.1180
FTm	0.0025	0.0030	0.0018
2-ptM	0.2302	0.1731	0.2325
3-ptM	0.2218	0.2565	0.3014
2-ptm	0.0229	0.0082	0.0162
3-ptm	0.0155	0.0239	0.0128
OREB	0.0030	0.0096	0.0020
STL	0.0038	0.0074	0.0093
TOT	0.0037	0.0107	0.0077
TOV	0.0978	0.0823	0.1243
V	0.0001	0.0002	0.0006
AST	0.1378	0.1617	0.1270
PF	0.0151	0.0282	0.0238
TF	0.0000	0.0004	0.0014
UF	0.0000	0.0000	0.0000
HA	0.0009	0.0034	0.0024
BLK	0.0009	0.0018	0.0029

## Discussion

This study primarily investigates basketball game pace by analyzing possession duration through clustering techniques. While clustering captures the temporal dynamics of game pace, event-level variables (e.g., turnovers, missed shots) are subsequently analyzed using machine learning models to determine their specific contributions within each pace segment. This dual-layered analysis bridges the gap between overall game rhythm and individual game events, offering a more holistic understanding of game pace dynamics.

### Inner pace: The relative fast and slow segments within a game

In this study, we employed a sliding window approach to analyze sequences of five or more consecutive possessions, using the clustering results of possession times as thresholds. This allowed us to divide the game into three different frequency segments: High-Frequency Possession Transition Segment (HFS), Low-Frequency Possession Transition Segment (LFS), and Normal-Frequency Possession Transition Segment (NFS). The decision to select sequences of five or more consecutive possessions as the standard for segmentation was based on practical considerations—game pace must persist for a certain duration to be considered effective. This approach contrasts with previous research, which often categorizes games as fast- or slow-paced based on the total number of possessions in a single game [26–28]. We believe that such a broad classification might not be appropriate. In NBA games, a typical game consists of over 200 possessions [10,27], and labeling an entire game as fast-paced implies that every possession within that game is tagged as fast-paced. In reality, the pace of a game fluctuates and is influenced by various factors, such as team style, tactical strategy, on-court events, and possibly random factors.

From a practical standpoint, HFS can be identified as phases of the game where the offense-defense transition frequency is quite rapid, with an average possession time of less than 9 seconds. Conversely, LFS represents phases where the transition frequency is slower, with an average possession time exceeding 18 seconds. NFS includes possessions that either do not fall into the HFS or LFS categories or have an average possession time between 9 and 18 seconds.

### The temporal characteristics of different frequency segments

Overall, HFS accounts for the fewest possessions in the game, followed by LFS, with more than half of the possessions categorized as NFS. This distribution aligns with the dynamic of basketball, where the internal rhythm of the game frequently oscillates between faster and slower possession segments [13,29]. However, we observe that different frequency segments exhibit characteristics that change over one game timeline. As shown in the upper right quadrant of Fig 3, for HFS, there is a trend of increasing duration within a single quarter, reaching its peak in the final three minutes of the quarter. Similarly, across the four quarters of the game, the duration of HFS progressively increases, peaking in the fourth quarter. This trend suggests that the occurrence of fast-paced segments tends to increase as the game progresses. This phenomenon may be related to the tactical arrangements, physical condition, and game context in both the early and later stages of the game and quarters [30–32]. In the early stages of the game and each quarter, teams are more likely to execute predetermined, relatively complex tactical maneuvers to gain a strategic advantage [6,31]. Additionally, due to the short rest periods between quarters and the relatively fresh state of the players at the beginning of each half or quarter, their energy levels are higher, defensive intensity is greater, and turnover rates are lower, making it difficult to create fast-break opportunities [32]. On the other hand, in the later stages of the game and quarters, as physical stamina wanes, it becomes harder to maintain high defensive intensity and low turnover rates, leading teams to opt more frequently for fast breaks and transition offenses [13]. This could also be linked to the players’ psychological states; as their stamina decreases, they may become more prone to rushing plays, with star players relying more on their individual abilities to get a quick isolation [33,34]. In some critical situations, there may also be rapid offense-defense transitions, such as when a trailing team urgently needs to score quickly to save time as the game approaches its end. During the final moments of a quarter or in crucial moments of the game, teams may also intentionally foul for strategy purpose [35]. These factors might partly explain why the predicted HFS duration peaks in the final three minutes of the fourth quarter.

The Low-Frequency Segment (LFS) exhibits different temporal characteristics compared to the High-Frequency Segment (HFS). In terms of total duration, the predicted duration of LFS also shows an overall increasing trend as the game progresses, with the fourth quarter having the highest predicted LFS duration, approaching 40%. This suggests that the proportion of slow-paced possessions increases as the game advances, possibly due to deliberate tactics taken by the teams to slow down the game pace [13]. Consecutive fast-paced transitions may lead to situations like a decline in shooting accuracy, an increase in fouls, turnovers, or a widening point difference. In such scenarios, coaches might use substitutions, timeouts, or other strategies to slow down the game pace to ensure effective use of possessions [36]. Additionally, in the fourth quarter, the leading team might adopt a slower offensive pace to gain an advantage in managing the remaining game time [35]. Contrary to our expectations, the beginning of each quarter has the lowest predicted proportion of slow-paced possessions. At the start of a quarter, due to pre-game planning and the break between quarters, coaches might be expected to employ a variety of tactical actions to create better shooting opportunities [37]. However, our results suggest that this might not be the case in reality. Meanwhile, in the middle of each quarter, typically within the 3–9 minute range, the game often experiences a slower pace. This characteristic of fast game pace at the beginning of the quarter and slow pace in the middle of the quarter may be related to the offense and defense strategy of both sides and the status of players, but the detailed explanation is worthy of further study. Towards the end of each quarter, there is a noticeable decline in the predicted LFS duration, which could be attributed to the significant increase in HFS during the final three minutes of the quarter.

The predicted duration of the Normal-Frequency Segment (NFS) shows a decreasing trend as the game progresses and as time advances within each quarter. This clearly indicates that as time progresses, the game is more likely to feature sustained fluctuations between fast and slow paces, rather than simply appearing in a chaotic rhythm [38,39].

### Comparison of indicator importance across different segments

In previous research on KPIs in performance analysis, distinctions are often made based on the differences in indicators between the winning and losing teams, thereby highlighting the importance of different indicators [5,6,11,12]. In this study, compared to traditional methods, LightGBM is better at handling nonlinear relationships and is more tolerant of collinearity issues within the feature values. The results also show that LightGBM has good prediction accuracy and robustness. Additionally, by using SHAP, the importance ranking of the indicators in the prediction process is quantified, helping coaches and athletes better understand the priority and significance of different indicators.

The previous discussion highlighted the temporal characteristics of different frequency segments, making it essential to differentiate the key performance indicators (KPIs) that contribute to winning in each segment. This can help coaches develop more effective strategies based on the dynamic phases of the game. As shown in Figs 4 and 5, the top six indicators significantly impact the prediction of net points in HFS, LFS, and NFS. Overall, 3-point makes (3-ptM), 2-point makes (2-ptM), free throws made (FTM), defensive rebounds (DREB), assists (AST), and turnovers (TOV) are the most important indicators for predicting net points across all three segments. These results are consistent with previous research, reaffirming the overall importance of these metrics in basketball games [5,29,40].

However, the ranking of indicator importance in LFS and NFS is almost identical, while significantly different from that in HFS, which warrants special attention. In both LFS and NFS, 3-ptM emerges as the most crucial indicator in terms of predictive validity and the number of samples affected. Teams in high-level basketball competitions today place great emphasis on players’ three-point shooting ability. Successful three-point shots not only provide a scoring advantage but also boost the team’s momentum [37,41]. The rankings of 2-ptM and DREB differ between LFS and NFS, primarily in terms of the number of samples impacted, rather than predictive validity (with DREB generally having higher predictive validity than 2-ptM). The “Moreyball” theory has influenced NBA gameplay in last two decades, suggesting that in slow-paced games, two-point shots might not be the best option, as players may prefer to end possessions with a three-point attempt [42–44]. However, since the success rate of three-point shots is lower than that of two-point shots, the importance of rebounding becomes more pronounced [42,43]. While assists (AST) do not directly result in scoring, they reflect the team’s organizational ability and the level of coordination among players. AST ranks fourth across all three types of games, indicating that team organization and player coordination have a similar level of impact regardless of the game type [3]. FTM and TOV rank fifth and sixth, respectively, in the importance of indicators in LFS and NFS, demonstrating that maintaining free-throw accuracy and reducing turnovers are also crucial in these types of games [29].

In conclusion, LFS and NFS games show a high degree of consistency in terms of the predictive validity of indicators, with only slight differences in the importance of DREB and 2-ptM. Coaches should focus on maintaining these key indicators in both slow-paced and normal-paced games while prioritizing DREB and 2-ptM as necessary.

In HFS games, the importance of free throws made (FTM) is the highest, indicating that FTM plays a significant role in predicting game outcomes in many matches. This is markedly different from the other two frequency segments. It suggests that during rapid transitions, players may have more opportunities to get free throws, and their ability to make free throws under high physical fatigue becomes crucial [32,44]. This is especially true in the final stages of the fourth quarter, where consecutive fouls and free throw situations occur frequently, and the player’s mental stability is key to ensuring successful free throws [35,45,46].

Additionally, in the fast-paced segment, the importance of 3-point makes (3-ptM) is greater than that of 2-point makes (2-ptM), which differs from the expectation that fast-paced transitions would more often conclude with a two-point shot. On one hand, this highlights the trend in modern basketball, where the pace of the game is accelerating, and the frequency of outside shooting continues to rise. On the other hand, it underscores the growing importance of players’ ability to shoot three-pointers during rapid transitions [41,47].

Turnovers (TOV) are slightly more important in HFS compared to LFS and NFS, indicating that minimizing turnovers yields higher benefits during fast-paced segments [36]. Lastly, it is worth noting that the importance of defensive rebounds (DREB) ranks only sixth in HFS. This suggests that compared to LFS and NFS, DREB is less successful in predicting net points in the fewer samples from HFS. This might be related to the higher uncertainty of rebound contests during fast-pace game, where defensive players may not be able to position themselves in time [42].

For indicators ranked lower in importance, we cannot conclude that they have no impact on the game. For example, home-court advantage and familiarity with the home environment may enable teams to perform better under certain game pace conditions, while timeout called by coach could influence the game pace. However, these effects are relatively less significant compared to the indicators discussed earlier. Future research could explore the influence of these other indicators on game pace dynamics from a multidimensional perspective.

In summary, it is crucial for coaches and related professionals to recognize the temporal characteristics of fast and slow frequency segments during a game, as this can deepen their understanding of the dynamic nature of offensive and defensive transitions. Additionally, understanding the differences in the importance of indicators across different segments allows coaches and players to develop better strategies, ultimately enhancing their chances of winning the game.

For the sake of rigor, we acknowledge several limitations of this study: First, the findings may not be generalizable to other basketball leagues (e.g., FIBA, NCAA), as different leagues’ rules, game durations, and playing styles could significantly influence the dynamic changes in game pace. Second, the exclusion of overtime data was primarily for analytical consistency. However, overtime represents a unique and critical phase of the game, involving player fatigue, tactical adjustments, and outcome predictions. Future research could focus on pace dynamics during overtime and their relationship to game outcomes. Lastly, due to the challenges in data collection, while this study observed an increase in high-frequency segments toward the end of the fourth quarter, it did not analyze the impact of player fatigue on game pace. Future studies are encouraged to incorporate physiological data (e.g., heart rate, running distance) for a more comprehensive analysis.

## Conclusion

This study provides a comprehensive analysis of game pace dynamics in basketball, highlighting the importance of distinguishing between high, low, and normal-frequency possession segments. By examining these segments across multiple game phases, we uncovered unique temporal characteristics, with fast-paced segments increasing in frequency as the game progresses, particularly towards the end of quarters. This reveals how game intensity escalates in later stages, driven by tactical, physical, and psychological factors. Conversely, slow-paced segments also increase but reflect strategic attempts to manage the game, particularly in the fourth quarter, when teams often slow down to control the clock. The findings demonstrate that understanding these pace fluctuations offers coaches and analysts a strategic edge, enabling them to refine tactics based on the pace dynamics of each game segment. Ultimately, our research emphasizes the nuanced nature of basketball game pace and offers insights that can enhance performance prediction and strategy optimization.

## Future Research

This study employed fixed thresholds (<9 seconds for fast pace, > 18 seconds for slow pace) derived from clustering and sliding window methods to identify pace frequency segments. While this approach provides generalizable insights into game pace dynamics, it may not fully capture team-specific differences in tactics, player configurations, and strategies. Future research could explore adaptive thresholds using techniques such as hierarchical clustering, multilevel regression, or mixed-effects models to better account for these variations.

Additionally, incorporating external contextual factors—such as home-court advantage, referee decisions, injuries, and rest days—could provide a more comprehensive understanding of their impact on game pace. Leveraging larger datasets or integrating contextual variables into machine learning models could enhance the robustness and applicability of findings across different scenarios.
